# Mechanisms of Therapeutic Resistance and Recent Advances in Glioblastoma Treatment

**DOI:** 10.1111/imcb.70144

**Published:** 2026-07-03

**Authors:** Emerson Achari, Farah Ahmady‐Nield, Amit Sharma, Ingo G. H. Schmidt‐Wolf, Jarek Maciaczyk, Rodney B. Luwor, Adrian A. Achuthan

**Affiliations:** ^1^ Department of Medicine Royal Melbourne Hospital, The University of Melbourne Parkville Victoria Australia; ^2^ Fiona Elsey Cancer Research Institute Ballarat Victoria Australia; ^3^ Federation University Australia Ballarat Victoria Australia; ^4^ Department of Integrated Oncology, Center for Integrated Oncology (CIO) University Hospital Bonn Bonn Germany; ^5^ Department of Neurosurgery University Hospital Bonn Bonn Germany; ^6^ Department of Surgery Royal Melbourne Hospital, The University of Melbourne Parkville Victoria Australia

**Keywords:** glioblastoma, hypoxia, immunosuppression, immunotherapy, tumor microenvironment

## Abstract

High‐grade central nervous system cancers incur a significant burden of care on society. The combination of therapeutic resistance and high mortality makes it both a challenging target and a devastating diagnosis. Of these, one in two is characterized as glioblastoma (GBM) with a median survival rate of only 13.5 months with the current standard of therapy. Modern interventions, such as PD‐1 and CTLA‐4 checkpoint inhibition and autologous CAR T cell delivery, remain stymied by both the difficult nature of drug delivery to the brain and the inherent immunosuppressive tumor microenvironment. However, recent advances in the characterization of GBM have unveiled promising new therapeutic avenues aiming to target and eliminate the tumor. In this review, we summarize the mechanisms through which GBM is initiated, localized, and eludes therapy responses and provide an update on recent advances made within this therapeutic space to overcome GBM‐mediated immunosuppression. We also discuss the challenges with current and next generational treatment strategies before finally exploring the landscape of potential future therapeutic targets.

## Graphical abstract

Three interconnected processes in glioblastoma (GBM) biology: initiation, stratification, and immunosuppression. *1) Initiation:* GBM initiation is proposed to occur through migration of TERT mutation‐bearing neural stem cells from the subventricular zone to the frontal and temporal cortices, where a subsequent oncogenic transformation drives their conversion into proneural glioblastoma stem cells. *2) Stratification:* advances in spatial transcriptomics have enabled stratification of intratumoral heterogeneity based on the relative spatial expression of mesenchymal, proneural, classical, astrocytic, and immune gene expression signatures, distributed along a decreasing hypoxia gradient radiating from the necrotic core. *3) Immunosuppression:* within the tumour microenvironment, GBM exploits both contact‐dependent mechanisms and contact‐independent mechanisms to drive polarisation and exhaustion of infiltrating immune cells toward a tumour‐tolerant phenotype.

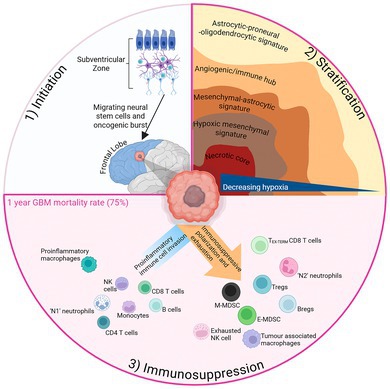

## 1 Introduction

Historically, the notion of extensive immunological activity within the brain has been a controversial affair. Evidence from the early 1900s, whereby predominantly epithelial tissue transplanted from skin to the anterior chamber of the eye and cerebellum of rabbit and rat models resulted in prolonged graft survival, suggested a limited capacity of immunological activity within the brain [1, 2]. This reinforced prior research by Paul Ehlrich [3] who showed that blood vessels within the brain restricted the movement of macromolecules through the formation of tight junctions by blood vessel endothelium thus isolating the brain from immunological activity, called the Blood Brain Barrier (BBB).

By marrying the BBB to graft studies, the exploration of immunology within the brain effectively stalled. While rapid progress was made in exploring the complex interactions between the immune system and tissue in various parts of the body, no such concurrent expansion was seen in the brain. This was the status quo until recently, where advances in technologies, such as RNA sequencing, flow cytometry, tissue microarray analysis, and better in vivo imaging techniques, warranted reinvestigation of this supposed ‘immune privileged state’. This resulted in the discovery of significant but highly restricted immune cell presence within the brain localized to barrier tissues such as the meninges, perivascular spaces, and choroid plexus [4, 5, 6]. Today, neurodegenerative conditions, such as Parkinson's disease and Alzheimer's disease, have been linked to dysregulated immune function [7, 8, 9]. Increased cytotoxic activity of meningeal CD8^+^ [10, 11] and Th_1_ and Th_17_ CD4^+^ T cells [12, 13, 14], for example, was found to drive pathogenesis in Parkinson's disease, with initiation mediated through decreasing Treg abundance at early stages [14]. Alzheimer's disease follows this interaction with CD4 Treg dysfunction linked to disease progression. This has consequently led to the application of Immunotherapeutics targeted at reestablishing T cell balance which are showing improvements in preclinical models [15, 16].

Comparatively, high‐grade CNS tumors remain largely unaffected by immunotherapeutic approaches. Among these, GBM is particularly notable, accounting for half of these tumors alone [17]. At present, the best standard of care for GBM is concurrent chemotherapy, namely Temozolomide (TMZ), and radiotherapy after maximal safe resection, a treatment that has not functionally changed since its inception in 2005 by Stupp et al. [18]. However, the highly diffuse nature of the tumor makes complete resection difficult where recurrent tumors may form in inoperable locations within the brain, and the adoption of resistance mechanisms to both radiotherapy and chemotherapeutic agents, such as TMZ, is common [19, 20, 21, 22, 23, 24]. This is typically associated with an epithelial to mesenchymal shift (proneural to mesenchymal subtype shift) with the tumor and ultimately resulting in the fatal nature of the tumor even with treatment, where the median survival hovers around at a dismal 13.5 months [25].

Exploration of the Tumor Microenvironment (TME) reveals a self‐reinforcing immunosuppressive state enriched in anti‐inflammatory immune cell populations, such as myeloid‐derived suppressor cells (MDSC), anti‐inflammatory monocyte‐derived macrophages (MDM) and CD4^+^ Treg cells which not only drive this immunologically cold nature but provide treatment resistance and promote invasiveness of the tumor. Recently, more modern immunotherapeutic interventions, such as checkpoint inhibition (e.g., anti‐PD1 and anti‐CTLA‐4) and engineered effector cells (e.g., Cimeric Antigen Receptor (CAR) ‐T and NK cells) have been applied to reinvigorate the antitumor response. Despite their efficacy in a broad range of cancers within the body, they have remained frustratingly ineffective in the treatment of GBM [26, 27, 28]. Therefore, the current landscape of GBM research is focused on the interrogation of the immunosuppressive TME for the development of more effective therapies [29]. To that end, studies have recently begun harnessing techniques, such as single cell RNA sequencing [30] and tissue microarray analyses [31], both to stratify the GBM TME as well as devise more advanced therapeutic interventions by interrogating the GBM and immune cell directed immunosuppressive pathways. Broadly, this review will serve as an update on the identification and categorization of GBM based on RNA sequencing and spatial transcriptomics as well as identifying key influences on immune cell populations driving this immunosuppressive state before finally exploring how the interrogation of these pathways is driving the development of ever more effective treatment for GBM.

## 2 Diagnosis

GBMs are typically diagnosed through T1 or T2 weighted magnetic resonance imaging (MRI) in patients in their 60s [17, 32] presenting with neurological symptoms, such as loss of visual acuity, seizures, headaches, and loss of balance, depending on the site of tumor formation [33, 34, 35, 36]. Tumors are typically 4–6 cm [33] on diagnosis and are predominantly located within supratentorial white matter tracts in either the frontal or temporal lobes [33, 36, 37]. Histologically, GBMs are identified by the presence of a necrotic core enclosed within a single layer of elongated cells called the pseudopalisade layer [38]. The invasive edge of GBM tumors is highly irregular and could appear as hypointense or hyperintense dependent on the T1 or T2 weighting of MRI, respectively [39]. The 2021 WHO classification of CNS tumors permitted the diagnosis of GBM based on molecular subtyping of widely recognized markers [40]. These are: gain of chromosome 7 and loss of chromosome 10; EGFR amplification, particularly resulting in expression of EGFRvIII and/or TERT promoter mutations resulting in sustained expression of the transcriptase. Based on the 2021 classification, isocitrate dehydrogenase (IDH) mutational status is now considered a distinguishing marker between astrocytoma (IDH mutant) and glioblastoma (IDH wildtype). Finally, O‐6‐methylguanine‐DNA methyltransferase (MGMT) methylation status is also explored where decreased methylation of MGMT promoter region is associated with increased efficacy of TMZ‐induced DNA lesion repair and is thus indicative of TMZ sensitivity [41].

## 3 Stratification of the GBM Tumor

**FIGURE 1:**
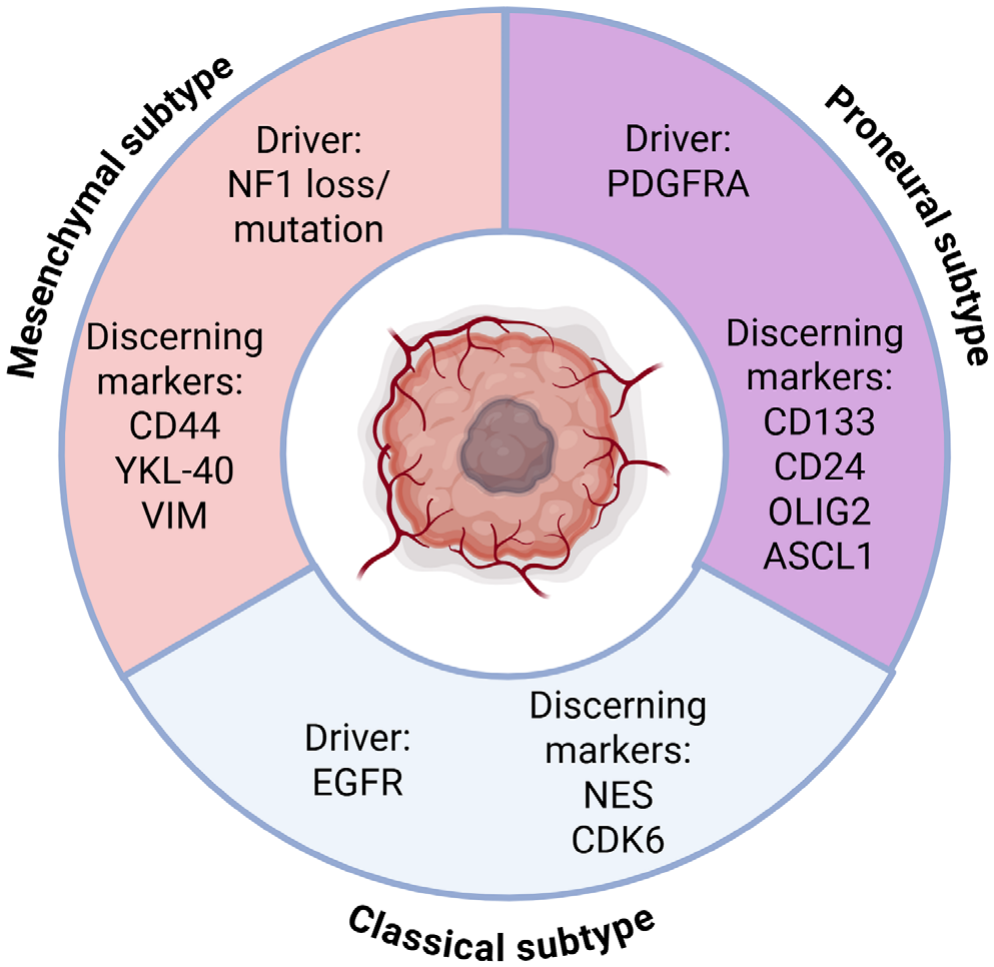
Subtypes of GBM. Schematic overview of the most prescribed distinguishing expression profiles of the molecular subtypes of GBM as defined by Wang et al. [42]. Mesenchymal subtype is mainly driven by mutation or loss of *NF1* and is characterized by expression of *CD44*, *YKL‐40*, and *VIM*. Proneural subtype is driven by *PDGFRA* overexpression and displays *CD133*, *CD24*, *OLIG2*, and *ASCL1* marker expression. Classical subtype is driven by *EGFR* overexpression (including *EGFRvIII*) and is distinguished by *NES* and *CDK6* expression.

While the molecular diagnostic markers provide some form of stratification of GBM based on their presence or absence, the inherent heterogeneity of the tumor TME has fueled further attempts to stratify GBM based on subtype marker expression. Today, there are three widely recognized subtypes of GBM based on differential gene expression of a subset of 20 genes as defined by Wang et al. [42] identified from the cancer genome atlas (TCGA). These are proneural (TCGA‐PN) defined mainly by high expression of *PDGRFA*; classical (TCGA‐CL) defined by high expression of *EGFRvIII*; and mesenchymal (TCGA‐MES) defined mainly by overexpression of angiogenic markers such as *VEGF* as well as frequent inactivating mutations of *NF1* (Figure 1).

Proneural subtype tumors were historically considered more treatable owing to the original inclusion of *IDH* mutations within the classification, thus resulting in the production of the oncometabolite D‐2 Hydroxyglutarate (D‐2HG) which hyper‐methylates the tumor, reducing its invasiveness [43, 44, 45]. However, the 2021 WHO reclassification of CNS tumors recharacterized *IDH* mutant bearing glioblastomas into oligodendroglioma or astrocytoma, dependent on the presence or absence of 1p/19q chromosomal codeletion [40], thus removing its utility as a proneural tumor marker and shifting survival toward a less favorable outcome [46, 47].

Mesenchymal tumors lie juxtaposed to their proneural and classical counterparts. Instead of adopting a broadly immunologically cold landscape, these tumors are generally characterized by abundant immune cell infiltration [48, 49, 50], resulting in a highly heterogenous and diffuse phenotype while remaining the most aggressive among the three [48]. The nature of this immune infiltration has widely been considered immunosuppressive [48, 49, 50]. Indeed, either inactivation or deletion of the tumor suppressor gene *NF1*, which negatively regulates the *RAS‐*cyclic *AMP* pathway to reduce cell growth [51], has been shown to both induce the onset of mesenchymal GBM as well as to drive a proportional increase in the aggregation of immunosuppressive tumor‐associated macrophages (TAM) to the TME [42, 52]. However, *NF1* mutation is not essential for the maintenance of the mesenchymal subtype, whereby mutation of the marker can only be correlated to approximately 30% of mesenchymal cells [42, 52]. This suggests that while *NF1* inactivation is a key mechanism in establishing the mesenchymal subtype, it cannot fully encapsulate the formation and maintenance of this immunosuppressive state. Instead, a broad network of interconnected pathways has since come to light which reinforce the immunosuppression [53, 54].

**FIGURE 2:**
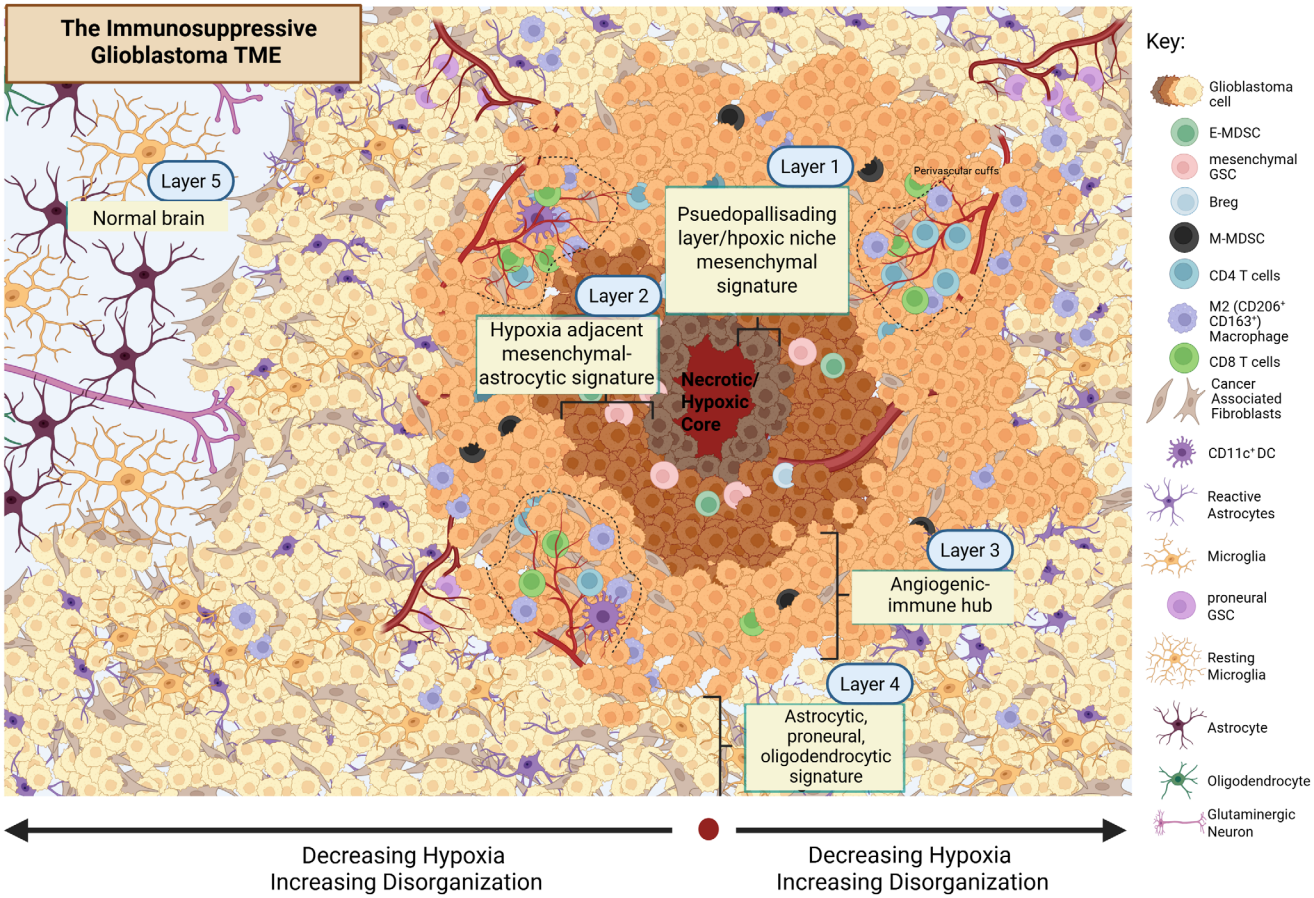
Schematic of an immunosuppressive GBM TME, stratified through Greenwald et al.'s layers, and mapped to immune populations. Figure shows decreasing spatial organization with a decreasing hypoxia gradient from the tumor core outward, based on layers described by Greenwald et al. [55]. Layer 1 is hypoxic niche, Layer 2 is hypoxia adjacent, Layer 3 is angiogenic hub, Layer 4 is astrocytic, proneural, oligodendrocytic signature, and Layer 5 is normal brain parenchyma. GSCs are spatially (Layer 1 vs. 4) defined based on their proneural vs mesenchymal origin. T cell infiltration is generally restricted to the perivascular cuffs in Layer 3. Vasectasia in blood vessels seen in Layer 2, but not evident in Layer 3 onward based on the presence or absence of mesenchymal GSC and CD31^+^ expression. Layer 5 is normal brain parenchyma enriched in glutaminergic neuronal activity.

Studies harnessing spatial transcriptomic techniques suggested that part of the tumor heterogeneity could be explained by spatially distinct tumor layers composed of overlapping GBM subtype signatures [30, 46, 55]. Effectively, answering the findings of co‐expression of these subtype gene signatures within patient tumor samples. Chief among these was Greenwald et al. [55], who used single cell RNA sequencing to cluster cells into ‘spots’ of similar gene expression, the greater the aggregation of similar spots, the higher the coherence of the tissue. The study proposed a layered model (Figure 2), where the central tumor mass predominantly consisted of a mesenchymal hypoxia dependent signature (Layer 1) that radiated outward toward a mesenchymal hypoxia independent signature (Layer 2) bordered by an angiogenic immune hub (Layer 3) and coinciding with glial microtube formation followed by a proneural or oligodendrocytic signature (Layer 4), indicating the invasive edge and finally reaching normal brain parenchyma [55]. This effectively married the cancer subtypes into structurally distinct regions of the *same tumor* rather than as discrete tumors, a factor that could not have been effectively identified by bulk RNA sequencing alone [56, 57]. Secondly, this organization was suggested to be highly correlated with decreasing hypoxia from the mesenchymal core outward (Figure 2). This further reinforced that decreased effector function as a consequence of hypoxia within GBM was a characteristic of mesenchymal GBM and further reinforced the influence hypoxia plays within its microenvironment.

GBM have long been known to harbor a subset of glioblastoma stem cells (GSCs) that serve as a self‐proliferative cellular reservoir for the generation of diffuse and highly heterogenous tumors [58]. The presence of these cells has been negatively correlated with patient survival due in part to their capacity to establish an immunosuppressive tumor microenvironment [54, 58, 59, 60]. These GSCs also upregulate *STAT3*, which has been linked to enhanced stemness and immunosuppressive functions [61].

**FIGURE 3:**
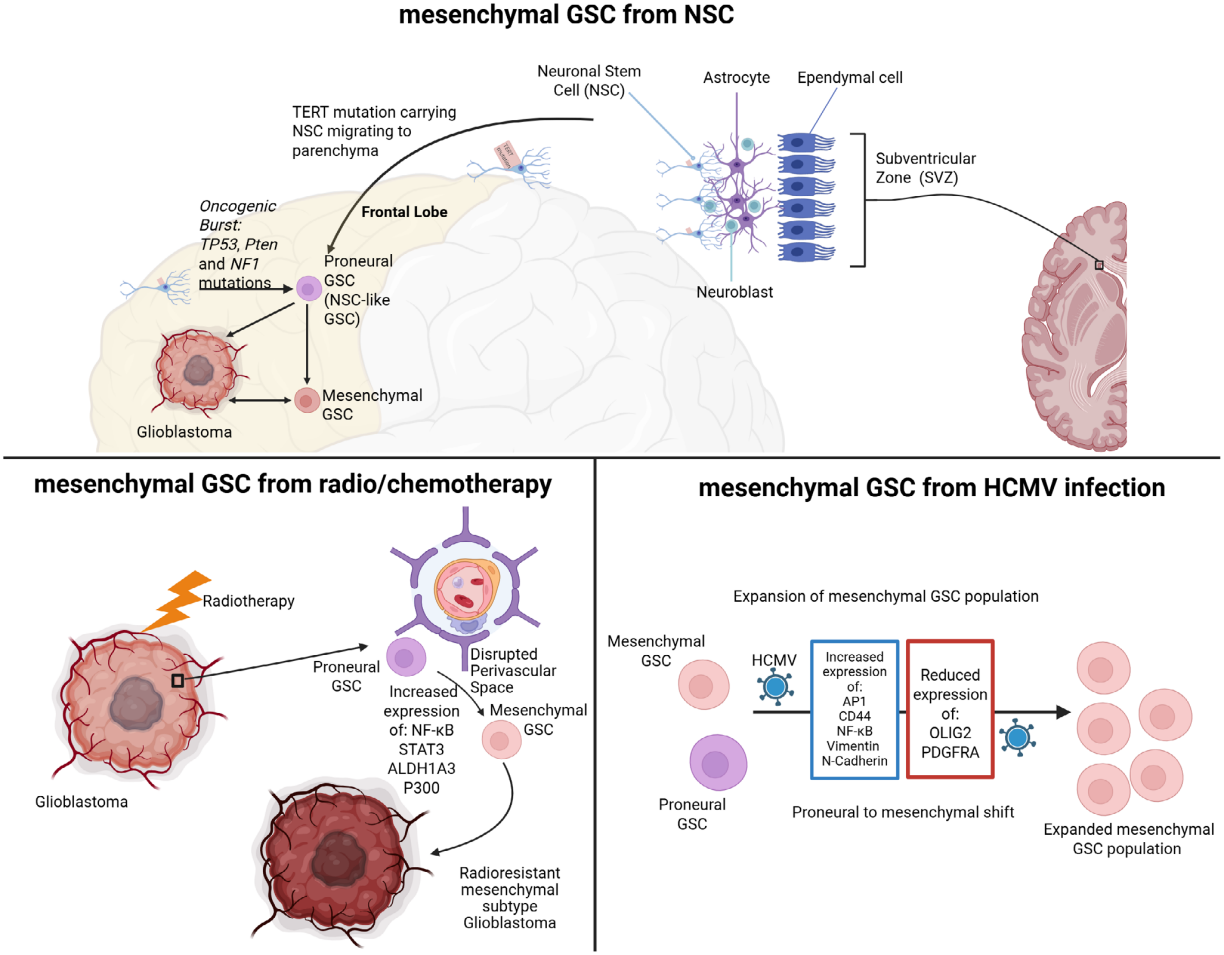
Mechanisms of formation of mesenchymal glioma stem cells (GSC). The upper panel depicts classical formation through differentiation from neural stem cells (NSC) bearing TERT mutations as they migrate from the subventricular zone (SVZ) to the frontal lobe brain parenchyma where an oncogenic burst of *NF1*, *TP53*, and *Pten* mutations results in *GSC* formation. Lower left panel depicts a proneural GSC to mesenchymal GSC shift due to increased expression of the NF‐κB pathway, *STAT3*, *ALDH1A3*, and *P300* resulting in both a migration away from the perivascular space to the tumor core and the contribution toward a radioresistant tumor. The lower right panel depicts the emerging role of human cytomegalovirus (HCMV) in driving mesenchymal *GSC* formation *through* both inducing a proneural to mesenchymal shift of GSCs as well as expanding mesenchymal GSC populations through upregulation of mesenchymal subtype gene expression (e.g., *AP‐1*, *CD44*, *NF‐κB*, *Vimentin*, and *N‐Cadherin*) while downregulating proneural subtype gene expression (e.g., *OLIG2*, *PDGFRA*).

All three subsets of GBM harbor GSCs, which divide along complementary expression profiles to their respective tumors [30, 62]. The most extensively characterized of which is the proneural GSC, which is neural stem cell (NSC) like. Lineage tracing studies track their origin to the subventricular zone (SVZ), whereby oncogenesis appears to be influenced by preexisting *TERT* mutations. Intracranial injection of hNSCs bearing doxycycline‐inducible mutations in *TP53*, *Pten*, and *NF1* in NOD‐SCID mice resulted in gliomagenesis in an ‘oncogenic burst’ rather than a gradual accumulation of mutations at T2; however, *EGFR* overexpression was only found to occur at low frequency and as an early onset mutation [63]. NSCs bearing these mutations migrated out of the SVZ and into the dorsolateral caudal cortex (Figure 3). Once at the site, these cells differentiated into heterogeneous tumors regardless of subtype. A subset of cycling NSCs localized around blood vessels and expressed *OLIG2*, *KI67*, *SOX2*, *CD133*, and *CD24*, markers of the GSC cell population [63]. Astrocytic and oligodendrocytic transcription factors are upregulated in these NSC‐like GSCs, which are directly regulated by the expression of the master transcription factor AP‐1. Inhibition of AP‐1 reduces the stem cell phenotype through decreased *OLIG2* and *KI67* expression [63].

Mesenchymal GSCs lack *CD133* expression and instead upregulate *CD44* and *YKL40* [30, 62], the latter of which promotes tumorigenesis and confers Temozolomide resistance through inhibition of DNA Damage Responses (DDRs) specifically in unmethylated MGMT GSCs [20]. Unlike their proneural counterparts, the origin of GSCs in the mesenchymal tumor subtype is less clear. While most agree that mesenchymal GSCs are formed from proneural GSCs (NPC‐like GSCs) [48, 64], proneural to mesenchymal transition has been known to occur upon tumor recurrence, particularly in response to chemotherapy and radiotherapy intervention [65, 66]. Interestingly, human cytomegalovirus (HCMV) infection has also been noted to induce a proneural to mesenchymal transition through upregulation of *RIP2*, activating the NF‐κB pathway [67] as well as through collaboration with TGF‐β1, resulting in activation of the JNK pathway [68] (Figure 3). The localization of these GSCs remains distinct between subtypes, with proneural NSC‐like GSCs predominantly located within the perivascular regions at the tumor edge [69, 70] while mesenchymal GSCs are located within the hypoxic niche, typically within the pseudopallisading zone [30, 46]. Additionally, mesenchymal GSCs have extensive interactions with immune cells, facilitating the formation of the immunosuppressive environment in a complementary fashion to hypoxia‐induced gene expression [71, 72, 73, 74].

## 4 Mechanisms Driving Immunosuppression in the TME

The tumor microenvironment encapsulating the developing GBM is a complex and dynamic structure that changes with both development of the tumor and therapy intervention. Cells within the TME are highly interactive with the tumor itself and surrounding parenchyma, often driving tumoral growth as well as reaffirming the immunosuppressive state. As such, the TME can often be a self‐reaffirming structure, where infiltrating systemic cytotoxic immune cells adopt more tolerogenic profiles dependent on the extent of intra‐ and peri‐tumoral modifying factors. Where these factors can be generated from or influenced by preexisting tolerogenic cells. Within the GBM TME, these drivers of immunosuppression can be stratified into the complementary categories of either direct cellular interaction or hypoxia‐induced tolerogenic reprogramming.

### 4.1 Immunosuppression Up Close‐ Decreased Cytotoxic Activity Through Direct Cellular Interaction

A significant challenge to the survival of a developing tumor is cytotoxic activity conducted primarily by MHC class I restricted CD8^+^ T cells. In response, cancers often downregulate MHC class I expression in concert with an upregulation of the ligand PD‐L1 [75, 76]. Downregulation of the former prevents neoantigen recognition, while upregulation of the latter induces T cell exhaustion. Combination of these two strategies results in the avoidance of CD8^+^ T cell‐mediated tumor destruction.

**FIGURE 4:**
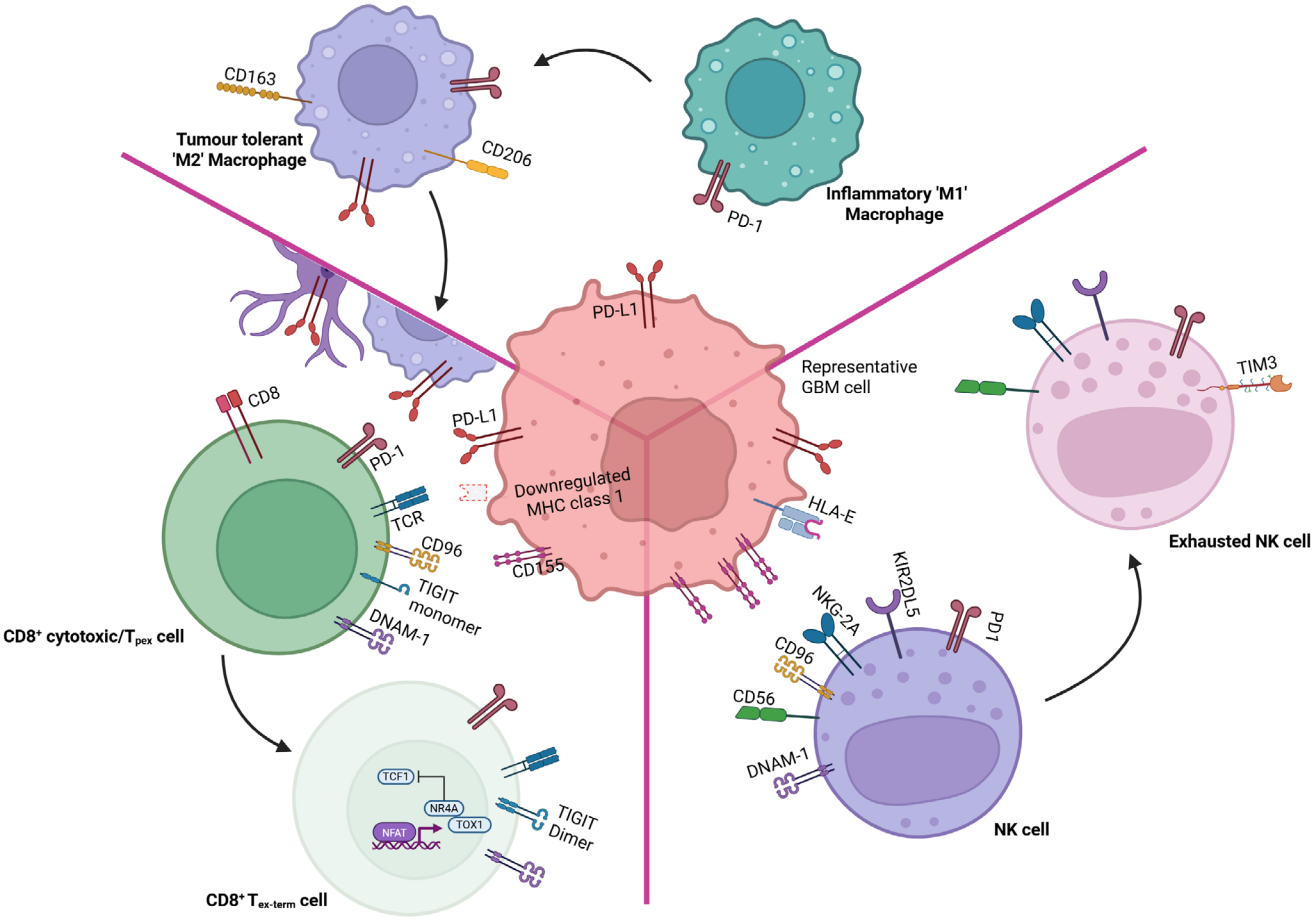
Mechanisms of contact inhibition employed by GBM cells to induce effector immune cell exhaustion or differentiation. Figure depicts direct immune cell interaction with GBM through inhibitory ligands and receptors which prevents effector immune cell response. Upper panel depicts PD1 expressing macrophages interacting with PD‐L1 expressing GBM which results in an ‘M2’ like polarization with expression of *CD206 and CD163* thus becoming more tolerogenic. Left panel depicts progenitor exhausted CD8+ T cells (Tpex) shifting to a terminal exhaustion state (Tex) through interaction with either GBM or ‘M2’ macrophage or reactive astrocyte expressed PD‐L1 and GBM expressed *CD155* with *PD‐1* and *TIGIT*, respectively, which drives *TOX1 and NR4FA* expression and inhibits *TCF1* expression. The right panel depicts NK cell exhaustion as a consequence of high expression of *CD155* on GBM cells interacting with *KIR2DL5 and HLA‐E* interaction with *NKG‐2A*.

GBM TME utilizes this strategy to great effect. PD‐L1 expression is widely expressed on GBM cells themselves [77, 78], reactive astrocytes [79], glioma‐associated macrophages [80], microglia [80], and carried within GBM‐derived extracellular vesicles (EVs) [81] suggesting an ‘immunosuppressive wall’ against CD8^+^ T cell activity (Figure 4). Furthermore, outside of immune interaction, expression of PD‐L1 on GBM cells has been linked to enhanced proliferation, migration, and invasive potential of these cells by upregulating the STAT3/IRAK2 pathway [77]. IRAK2 activated NF‐κB, which in turn drove production of the inflammatory IL‐6 cytokine. This induced both MDSC expansion and DC inhibition, further reinforcing the immunosuppressive state [77]. However, exhaustion within these CD8^+^ T cells is not an end state. Recent studies have shown that a subset of these cells can adopt a proliferative exhausted phenotype (T_pex_), denoted by expression of PD‐1^+^ CD39^−^ Slamf6^+^ TIM3^−^ TCF1^+^ TOX1^High^ Ly‐108^+^ CXCR5^+^, which differentiate into effector exhausted (T_ex‐eff_), denoted by: PD‐1^+^ CD39^+^ Slamf6^−^ TIM3^+^ TCF1^−^ TOX1^Low^ Ly‐108^−^ CXCR5^−^, before finally becoming terminally exhausted (T_ex‐term_), denoted by PD‐1^+^ CD39^+^ Slamf6^−^ TIM3^+^ TCF1^−^ TOX1^High^ Ly‐108^−^ CXCR5^−^ and losing all cytotoxic capacity [82]. The presence of these T_pex_ cells allows for cytotoxic activity in the face of high PD‐L1 expression within these tumors. PD‐1 checkpoint inhibition results in an immediate proliferative burst of T_pex_ cells with subsequent differentiation into T_ex‐eff_ cells [83, 84, 85].

Recently, such a CD8^+^ T cell stem‐like population was identified to exist within the GBM TME. Wang et al. [86] characterized an exhausted TIM3^+^ CD101^+^ proliferative CD8^+^ T cell population that differentiated into a further exhausted CD101^−^ subtype, which they termed CCT precursor and CCT cells, respectively. Both cell populations differed significantly in their intracellular pathways, with CD101^+^ cells showing extensive interaction with SPP1^+^ macrophages, a pro‐tumorigenic population known for promoting CD8^+^ T cell exhaustion and which subsequently diminished with PD‐1 blockade. Both TIM3 and CD101 have previously been included as markers of both T_ex‐eff_ and T_ex‐term_, which are TIM3^+^ CD101^+^ and TIM3^+^ CD101^−^, respectively [87] suggesting that these CCT precursor cells and CCT cells bear a strong similarity with T_ex‐eff_ and T_ex‐term_ cells (Figure 4).

As a companion to PD‐L1 expression, GBM cells upregulate CD155 [88] which preferentially binds to the inhibitory receptor TIGIT compared to the stimulatory CD226 [89] expressed on T_pex_ cells. This results in a shift from T_pex_ to T_ex‐term_ cells in the tumor, thus removing responsiveness to PD‐1 checkpoint therapies [90]. While the exact mechanisms for this transition are still debated, it is likely driven through AP‐1 independent translocation of NFAT to the cellular nucleus which then subsequently drives the transcription of *TOX1* and *NR4A* which in turn inhibit TCF1 expression [88, 91, 92] (Figure 4). It is unclear whether this mechanism of action is achieved through CD155 and TIGIT interaction or occurs independently through TCR chronic stimulation. Multiple studies have shown that inhibition of *TIGIT* rescues *TCF1* expression at the expense of *TOX1* and *NRFA2*, suggesting that *TIGIT* is in part responsible for the T_ex‐term_ phenotype [88, 93].

While PD‐1 expression is most associated with lymphocyte populations, a growing body of evidence is highlighting its importance within granulocyte myeloid progenitor derived cells in the TME [94, 95, 96, 97]. PD‐1 directly impairs IFNγ secretion through direct interaction with the JAK2/STAT3 pathway and promotes the differentiation of cells into tolerogenic ‘N2’ neutrophils, monocytic myeloid‐derived suppressor cells (M‐MDSC), and tolerogenic ‘M2’ macrophages (Figure 4), which in turn drive immunosuppression through suppressive cytokine secretion [94, 95]. Specific ablation of PD‐1 on monocytes results in a shift in macrophage polarization toward a proinflammatory ‘M1’ state [97]. This extends to T cell involvement, where an increase in effector memory (IFNγ^+^ IL‐17A^+^ IL‐10^+^) CD8^+^ T cells can be observed [94, 96, 97].

The downregulation of classical MHC class I genes (HLA‐A, HLA‐B, and HLA‐C) by cancer cells may avoid CD8^+^ T cell‐mediated immunosuppression but presents an opportunity for the activity of NK cell‐mediated destruction. To avoid this, GBM employs complementary inhibitory mechanism of CD155 overexpression and noncanonical HLA‐E loaded with peptides from HLA‐G or HLA class 1. Unlike CD8^+^ T cells, CD155 can induce NK cell cytotoxicity and IFNγ secretion through binding with the activating receptor DNAM‐1 and CD96 [98]. However, recent studies have shown that sustained overexpression of CD155 paradoxically downregulates DNAM‐1 potentially due to increased interaction with the inhibitory receptor KIR2DL5, subsequently impairing IFNγ secretion [99, 100, 101] (Figure 4). This parallels the interaction with CD8^+^ T cell exhaustion, an observation that is only reinforced by the expression of PD‐1 on NK cells in GBM, which induced exhaustion and NK cell‐mediated immunosuppression [102]. Direct interaction of PD‐1 expressing CD56^bright^ NK cells with proneural GSCs has been reported within the perivascular space where NK cytotoxic activity was modulated by CD155 expression [99].

Furthermore, NK cells express the inhibitory NKG‐2A and the immunostimulatory NKG‐2C membrane bound receptors which bind to the non‐classical HLA 1b marker, HLA‐E [103]. The frequency of this NK receptor expression varies with both the maturation state of the cell and the disease environment. In glioblastoma, both HLA‐E and NKG‐2A are highly upregulated in their respective cell types, triggering extensive NK cell immunosuppression (Figure 4). This interaction is particularly notable with proneural GSC cells (as with PD‐1 expression), where IFNγ and irradiation induced upregulation of HLA‐E has been reported [104]. The interaction of HLA‐E with NKG‐2A results in immunosuppression of NK cells and has been linked to the reversible exhaustion of the cell through inhibition of the PI3K/AKT pathway [105, 106].

### 4.2 Immunosuppression at a Distance‐ Hypoxia

Hypoxia is a widely recognized mechanism for driving tolerogenic immune activity due mainly through the upregulation of the hypoxia response genes of the HIF family. Several recent reviews already cover the role of this family of genes in hypoxia [60, 107, 108]. This section will thus focus on the less explored hypoxia‐related roles of *HMOX1* and *STAT3* predominantly in mesenchymal GBM.

Typically, hypoxia induces the upregulation of *STAT3* in an IL‐6 and IL‐10 dependent fashion through the JAK/STAT pathway. This in turn upregulates hypoxia inducible genes such as *VEGFA*, *HK1*, *HK2*, *PFKP*, *HILPDA* as well as *HMOX1* [109, 110]. While HIF‐1α stabilization is most prominently associated with hypoxic response [59, 60, 111, 112, 113, 114], the ablation of *STAT3* resulted in impaired immune cell response and angiogenesis, suggesting a fundamental role in hypoxia [109]. Furthermore, pericytes within the vasculature of the brain have been shown to upregulate *STAT3* earlier than *HIF‐1α* under hypoxic conditions [115], once again suggesting that STAT3 may be an earlier stage regulator of ischemic or hypoxic response [116]. However, interaction between *STAT3* and *HIF‐1α* is well documented [109, 116, 117], and thus it is likely that while cytotoxic factors may initiate *STAT3* activation, *HIF‐1α* maintains its function. From a clinical standpoint, GBM tumors with high expressed *HMOX1* or *STAT3* are linked to worse clinical outcomes [118, 119]. This expression profile greatly contributes to the broad tolerogenic immune landscape. For example, tumor infiltrating CD8^+^ T cells have been known to lose cytotoxicity and instead adopt an exhausted phenotype in mesenchymal tumors [120]. Subsequent Nearest Functional Connected Neighbor (NFCN) and CD3^+^ IBA1^+^ (*AIF1*) doublet analysis identified that close interaction with HMOX1^+^CD163^+^ macrophages and microglia may play a driving factor in this exhausted state. Functional depletion of these hypoxic myeloid cells resulted in increased expression of *GZMB* indicating restoration of effector function in an IL‐10‐dependent manner [121]. Additionally, HMOX1^+^ CD163^+^ monocytes have been subsequently shown to preferentially migrate to hypoxic mesenchymal cells where they subsequently upregulate CCL2 secretion, a lymphocytic recruiting chemokine [120, 121]. In line with Greenwald et al.'s [120] characterization of an immunological layer, the exhausted CD8^+^ T cell expression closely correlated with a mesenchymal‐astrocytic tumor signature.

*STAT3* upregulation has also been observed in tumor infiltrating Tregs [122], which contribute to the immunosuppressive phenotype predominantly through secretion of IL‐10 and TGFβ [123, 124]. These Tregs can potentially upregulate *HMOX1* production in response to oxidative stress [119]; however, studies have shown an antagonistic relationship between *HMOX1* expression and TGF‐β signaling [125, 126, 127]. Given that STAT3 is an upstream regulator of HMOX1, it is likely that *STAT3* upregulation rather than *HMOX1* expression is the prevailing pathway for hypoxia‐resistant Tregs in GBM. Additionally, *STAT3* activation is essential for the recruitment of Th_17_ cells [128, 129, 130, 131]. Here, infiltration has been shown to be a poor prognostic indicator in GBM [132, 133]. *STAT3* activation under the influence of TGF‐β has been shown to induce Th_17_ polarization [134]. This effect is magnified under hypoxia due to upregulation of HIF‐1α [135].

Within myeloid cells, the role of *STAT3* has been well described in driving anti‐inflammatory macrophage, neutrophil, and dendritic cell populations. Analysis of TCGA and the Chinese Glioma Genome Atlas (CGGA) showed upregulation of *HMOX1* within mesenchymal glioma. This upregulation correlated with increased M2 macrophage infiltration and increased expression of checkpoint markers TIM‐3, PD‐1, CD273, and HVEM. This resulted in decreased infiltration of CD4, NK, and T follicular helper cells [136]. Additionally, the *HMOX1* and *STAT3* expression were found to be coupled in these infiltrating M2 macrophages whereby *HMOX1* knockdown reduced CD163^+^ M2 macrophage infiltration. Neutrophils harbor a near identical phenotype, where *STAT3* upregulation has been linked to the anti‐inflammatory ‘N2’ state and impair type I IFN signaling [137, 138]. Neutrophil‐specific inhibition of *STAT3* resulted in increased expression of cytotoxic IFN‐γ^hi^Ki67^hi^granzyme B (GZMB)^hi^ perforin^hi^ population of CD8^+^ T cells specifically, with no effect on the CD4 T cell compartment [139]. Dendritic cells follow a similar trend where *STAT3* blockade in combination with whole brain radiation therapy (WBRT) induces tighter CD11c^+^ DC and T cell interactions which results in longer patient survival [140]. Additionally, all three myeloid populations have been shown to secrete IL‐10 and TGFβ [141, 142, 143], which have strongly been associated with the immunosuppressive milieu.

## 5 The Trajectory of GBM Treatment

In 2005, Stupp et al. [18] altered the GBM treatment landscape with a landmark study depicting the inclusion of the chemotherapeutic agent Temozolomide to the regimen of surgical resection and fractional radiotherapy (60 Gy in 30 fractions) which resulted in a significantly increased median survival rate of patients from 12.1 to 14.6 months. This approach would become the standard of care for GBM diagnosed patients. Unfortunately, since its implementation, there has been little widespread improvement in GBM treatment. While advances in the techniques of radiotherapy delivery such as targeted intensity modulated radiotherapy (IMRT) and volumetric modulated arc therapy (VMAT) have further improved the specificity of the RT delivery to limit RT mediated neurotoxicity [144], they have not been able to improve the median survival outcomes of patients, nor have they been able to reduce the high 90% recurrence rate [144]. To address this failure of therapeutic response, the modern landscape for GBM interventions is highly diverse, attempting to either refine the current standard of care or supplement its efficacy through immunotherapeutic and hypoxia targeting means.

### 5.1 Radiotherapy Techniques

Radiotherapy techniques such as proton therapy (PT) [145], boron neutron capture therapy (BNCT) [146] and carbon ion radiotherapy (CIRT) [147] are currently under investigation in early‐stage clinical trials but lack large‐scale randomized controlled trial data [144, 145, 146, 147]. These approaches permit highly targeted radiation to the tumor core, reducing the likelihood of radiation‐induced neurotoxicity (notable for PT) while simultaneously permitting a higher dose of radiation to the tumor site (BNCT dose: ~20Gy‐Eq per fraction) and can be used as a hypofractionation agent with the current standard of care [144].

### 5.2 Immunotherapies

While there has been a plethora of immunotherapeutic approaches for targeting GBM, the classical examples of the field remain checkpoint inhibition and CD8^+^ CAR T cell generation. The former encompasses anti‐PD‐1 therapies such as nivolumab [148] and pembrolizumab [149] as well as anti CTLA‐4 therapies such as ipilimumab [150] which predominantly target inhibitory receptors on CD8^+^ T cells. Such therapies have proven effective in solid tumors, such as melanoma [151] and NSCLC [152], but have remained poor in their effectiveness in GBM resulting in failed stage III clinical trials even with codelivery [153] (Table 1). A similar story is evident in CD8^+^ CAR T cells, which are engineered to avoid MHC restriction and target highly expressed cancer‐specific antigens, such as EGFRvIII, IL13Rα2, HER2, and B7‐H3, expressed by GBM cells [154, 155]. The site of delivery of CAR T cells (intravenous vs. intraventricular) influences the efficacy of therapy, with increased proximity to the tumor site increasing CAR T cell retention [154]. Unfortunately, this treatment approach suffers from poor retention in GBM, often resulting in exhaustion through widespread PD‐L1 interaction [156]. Attempts to circumvent this through codelivery with pembrolizumab or engineering of PD‐1‐deficient CAR T cells have not yielded significant improvements to patient median overall survival [157] (Table 1). As such, CAR T cell development remains mainly situated at preclinical and early clinical development stages [154]. Subsequent therapies such as oncolytic viruses like PVSRIPO targeting CD155 expression, oncolytic vaccines, and microRNA therapies are either preclinical or at early‐stage clinical trials and suggest promising pilot data, but larger randomized control trials would be required [158, 159].

**TABLE 1:** Overview of various therapeutic approaches for GBM in clinical trials.

Intervention type	ClinicalTrails.gov ID	Phase	Arms	Condition	Survival outcome
Checkpoint Inhibition	NCT02017717	Phase III: Randomized open label trial	Arm 1: Nivolumab (anti‐PD‐1 monoclonal antibody) Arm 2: Bevacizumab (anti ‐VEGF monoclonal antibody)	Recurrent Glioblastoma	12 months overall survival (OS) (as % of participants) Arm 1: 41.8 (95% CI: 34.7 to 48.8) Arm 2: 42.4 (95% CI: 34.9 to 49.6) 10 years and 5 months progress free survival (PFS) (months) Arm 1: 1.5 (95% CI, 1.5–1.6) Arm 2: 3.5 (95% CI, 2.9–4.6)
	NCT04396860	Phase II: Randomized open label trial	Arm 1: Radiation therapy + Ipilimumab + Nivolumab Arm 2: Radiation therapy + Temozolomide	Gliosarcoma; MGMT‐Unmethylated Glioblastoma	19.3 months PFS (months) Arm 1: 8.5 (95% CI: 7.1 to 10.4) Arm 2: 7.7 (95% CI: 6.5 to 8.5)
	NCT02794883	Phase II: Randomized open label trial	Arm 1: Durvalumab (anti‐PD‐L1 monoclonal antibody) Arm 2: Tremelimumab (anti CTLA‐4 monoclonal antibody) + Durvalumab Arm 3: Tremelimumab	Malignant Glioma; Recurrent Glioblastoma	24 months median OS (months) Arm 1: 7.5 (95% CI: 2.3 to 14.7) Arm 2: 13.2 (95% CI: 2.6 to 28.0) Arm 3: 7.9 (95% CI: 4.8 to 14.2) 24 months PFS (months) Arm 1: 3.1 (95% CI: 1.9 to 5.1) Arm 2: 4.4 (95% CI: 1.2 to 11.6) Arm 3: 3.2 (95% CI: 2.9 to 4.0)
CAR T cells	NCT05366179	Phase I: Dose escalation trial	Single Arm: CAR B7‐H3T cells infusion	Glioblastoma Multiforme	No results published
	NCT05474378	Phase I: Dose escalation trial	Single Arm: CAR B7‐H3T locoregional delivery	Recurrent Glioblastoma Multiforme	No results published
	NCT06186401	Phase I: Dose escalation trial	Single Arm: Anti‐EphA2/IL‐13Ralpha2 CAR (E‐SYNC) T Cells delivery	EGFRvIII+ Glioblastoma	No results published
	NCT05168423	Phase 1: Dose escalation study	Arms: CART‐EGFR‐IL13Ra2 Cells delivery	EGFR‐Amplified Recurrent Glioblastoma	No results published
	NCT03726515	Phase 1: Dose escalation study	Single Arm: CART‐EGFRvIII + Pembrolizumab (anti‐PD‐1 monoclonal antibody)	Newly Diagnosed, MGMT‐Unmethylated Glioblastoma	14 months median OS (months) 11.8 (90% CI: 9.2–14.2) 14 months PFS (months) 5.2 (90% CI: 2.9–6.0)
CIK cells	NCT06684899	Phase I: Cross sectional study	Arm 1: Patients with GBM Arm 2: Healthy controls	Glioblastoma	No results published
	NCT00807027	Phase III: Randomized open label trial	Arm 1: Temozolomide + Radiotherapy Arm 2: Temozolomide + Radiotherapy + autologous CIK cell infusion	Glioblastoma	46 months median OS (months): Arm 1: 16.9 (95% CI: 13.9 to 21.9) Arm 2: 22.5 (95% CI: 17.2 to 23.9) 46 months PFS (months): Arm 1: 5.4 (95% CI: 3.3 to 7.9) Arm 2: 8.1 (95% CI: 5.8 to 8.5)
Adenovirus	NCT02197169	Phase I: Randomized open label	Arm 1: DNX2401 (conditionally replicative oncolytic human derived adenovirus) delivered subcutaneously Arm 2: DNX2401 + IFNγ Arm 3: DNX2401 delivered via MEMs cannula	Recurrent Glioblastoma; Gliosarcoma	9 months OS (as % of participants): Arm 1: 44 Arm 2: 33 Arm 3: 40 18 months OS (as % of participants): Arm 1: 22 Arm 2: 22 Arm 3: 0

In the evolving landscape of GBM treatment, it is equally important to emphasize the contribution of adoptive cellular immunotherapy (ACI) approaches, which involve isolating immune cells from a patient or donor, expanding or engineering them outside the body, and reinfusing them to specifically target and eliminate cancer cells. Among these, cytokine‐induced killer (CIK) cells, which are ex vivo expanded lymphocytes with both T cell and natural killer (NK)‐like properties, have shown considerable promise for treating GBM. In clinical settings, phase III randomized trials in South Korea demonstrated that adding CIK cell therapy to standard TMZ chemoradiotherapy significantly improved progression‐free survival (PFS), although effects on overall survival (OS) were variable. Analyses focusing on pathologically pure GBM further indicated that CIK therapy may independently predict improved OS and PFS with an acceptable safety profile [160, 161]. Supporting these observations, an independent study reported that two of six patients with malignant glioma, including one with G34‐DHG (WHO grade 4), survived more than 20 years following local CIK cell immunotherapy [162]. Building on these encouraging clinical results, preclinical studies have explored strategies to enhance CIK efficacy. The combination of dendritic cells with CIK cells (DC‐CIK) has shown improved antitumor activity in GBM models, providing a potential alternative to conventional CIK therapy [163]. In addition, a novel immunocytokine αBC‐IL15 has recently been reported to promote T cell‐ and CIK‐mediated antitumor immunity via a non‐MHC‐restricted mechanism in GBM [164]. Furthermore, PPAR‐γ inhibition in GBM cells has been shown to potentiate CIK cytotoxicity, suggesting a promising combinatorial approach to overcome tumor resistance and improve therapeutic outcomes [165]. Collectively, these findings underscore the evolving potential of CIK‐based adoptive immunotherapy in GBM, highlighting both clinical feasibility and preclinical innovations that may enhance antitumor efficacy.

### 5.3 Hypoxia‐Targeting Therapies

Where immunotherapy targets cellular interactions, treatments focused on hypoxia target oxygenation flow to the tumor site. If immunosuppression is mediated through immune response to hypoxia, then treating hypoxia would inhibit tumor growth. While this approach works with solid tumors, such as head and neck squamous cell carcinoma (HNSCC), where the addition of a hypoxic radiosensitizer resulted in increased disease‐free survival and locoregional control [166], GBM is largely nonresponsive to hypoxia‐targeting therapies. Interventions are divided into two categories: The first is directly targeting angiogenesis, either through anti‐VEGF IgG antibodies such as bevacizumab [167, 168]; integrin αvβ3 and αvβ5 targeting cligenitide [169]; or directly increase oxygen perfusion through trans‐sodium crocetinate or myo‐inositol trispyrophosphate acting on plasma and hemoglobin, respectively [170, 171]. The second is directly targeting *HIF‐1α* and *HIF‐2α* gene expression through a variety of mRNA, protein, and miR inhibitors [172]. Unfortunately, clinical trials suggest that these therapies may not provide better patient survival in GBM [172, 173] (Table 2).

**TABLE 2:** Overview of bevacizumab and cligentide therapies targeting hypoxia in GBM.

Intervention type	ClinicalTrails.gov ID	Phase	Arms	Condition	Survival outcome
Bevacizumab	NCT00884741	Phase III: Randomized double blind placebo control trial	Arm 1: Radiation + Temozolomide + Placebo Arm 2: Radiation + Temozolomide + Bevacizumab	Newly Diagnosed Glioblastoma	Median overall survival (OS) (months); assessed at participant death: Arm 1: 16.1 (95% CI: 14.8 to 18.7) Arm 2: 15.7 (95% CI: 14.2 to 16.8) Progress free survival (PFS) (months); assessed at participant death: Arm 1: 7.3 (95% CI: 5.6 to 7.9) Arm 2: 10.7 (95% CI: 10.0 to 12.2)
	NCT01290939	Phase III: Randomized open label trial	Arm 1: Lomustine + Bevacizumab Arm 2: Lomustine (alkylating agent)	Recurrent Glioblastoma	48 months median OS (months): Arm 1: 9.1 (95% CI: 8.1 to 10.1) Arm 2: 8.6 (95% CI: 7.6 to 10.4) 48 months PFS (months): Arm 1: 4.2 (95% CI: 3.7 to 4.3) Arm 2: 1.5 (95% CI: 1.5 to 2.5)
Cligentide	NCT00689221	Phase III: Randomized open label trial	Arm 1: Cilengitide + Temozolomide + Radiotherapy Arm 2: Temozolomide + Radiotherapy	Newly diagnosed glioblastoma with MGMT promoter methylation	48 months median OS (months): Aim 1: 26.3 (95% CI: 23.8 to 28.8) Aim 2: 26.3 (95% CI: 23.9 to 34.7) 48 months PFS (months): Aim 1: 10.6 (95% CI: 8.3 to 13.4) Aim 2: 7.9 (95% CI: 5.9 to 12.5)
	NCT00813943	Phase II: Randomized open label trial	Arm 1: Cilengitide (2‐times Weekly) + Temozolomide + Radiotherapy Arm 2: Cilengitide (5‐times Weekly) + Temozolomide + Radiotherapy Arm 3: Temozolomide + Radiotherapy	Newly Diagnosed Glioblastoma and unmethylated MGMT promoter	44 months median OS (months): Arm 1: 16.3 (95% CI: 13.2 to 18.1) Arm 2: 14.5 (95% CI: 12.6 to 16.5) Arm 3: 13.4 (95% CI: 12.2 to 14.3) 44 months PFS (months): Arm 1: 6.4 (95% CI: 4.2 to 7.9) Arm 2: 7.5 (95% CI: 5.9 to 8.2) Arm 3: 6.0 (95% CI: 4.1 to 7.7)

Alternatively, targeting drivers of GSC formation such as STAT3, which is frequently upregulated in hypoxic conditions, has recently emerged as an attractive therapeutic target. This is made even more crucial with pSTAT3 upregulation correlated with TMZ resistance [174]. miRNA (miR) serves as both a driver of the immunosuppressed state as well as a potential therapeutic target. EV packaged miR‐30b‐3p and miR‐210‐3p for example are induced under hypoxic conditions through the transcriptional co‐activation of HIF1α and STAT3 from hypoxic (mesenchymal) GSCs. These mIRs promote GSC proliferation inhibit apoptosis through upregulation of BCL‐2 and TGF‐β1, respectively [21, 23]. The latter of which was found to induce morphological alteration as well as E‐cadherin, N‐cadherin and Vimentin expression, all suggestive of an epithelial to mesenchymal transition thus increasing both invasive capability and resistance to TMZ [23].

On the other hand, miR‐124 was found to act antagonistically to both miR‐30b‐3p and miR‐210‐3p by restricting glioblastoma growth and triggering apoptosis [175]. miR‐124 principally inhibits *STAT3* both within mesenchymal GSC cells as well as neighboring T cells in the TME [176, 177]. This dual action has been shown to concurrently result in the differentiation of these GSCs into a mesenchymal glioblastoma cell, as well as promote the formation of a proinflammatory TME though secretion of TNFα and IFNγ from CD8^+^ and CD4^+^ Th_1_ T cells, while inhibiting the proliferation of both Th_17_ and CD4^+^ Treg cell populations [176, 177]. While this may indicate that miR‐124 is effective in reversing the activation of *STAT3*, the expression of the miR is significantly impaired under the hypoxic conditions [175, 177, 178, 179, 180]. Spatial exploration of glioblastoma reinforces this view, whereby a greater reduction of the miR expression can be seen within the hypoxic pseudopallisading region of the tumor over the less hypoxic periphery [175]. miR‐124 is regulated by *REST* [178], which in turn is highly expressed within GSCs in response to hypoxia [181], suggesting a potential regulatory pathway. *MAPK14/p38α* [175], *TEAD1* [175], *SERP1* [175], *CDK4* [176] and *CDK6* [176] are all direct targets of the miR and all have roles in GSC proliferation, self‐renewal, and cell survival, providing a further therapeutic target to limit tumoral growth. Coadministration of miR‐124 and PD‐1 via a dual gene delivery system has shown to result in increased apoptosis and TMZ sensitization in an orthotopic GL261GBM derived model [176]. While no miR clinical trials are presently underway, direct inhibition of pSTAT3 has been achieved through the small molecule inhibitor WP1066 which is currently in Phase II clinical trial (NCT05879250). This therapy has been shown to improve antitumor immunity specifically by driving potent effector T cell activation and simultaneously impairing the CD4^+^ Treg contribution to the TME [182, 183]. Besides targeting hypoxia, alternative approaches for targeting GSCs remain varied and have recently been thoroughly explored [184, 185].

## 6 Limitations of the Current Approach and Treatment Opportunities in the Future

Despite significant investment into GBM treatment, there yet remains no clear therapeutic avenue through which patient survival can be prolonged. The reasons behind this are myriad and developing but often include an underestimation of the inherent heterogeneity of the GBM tumor and its environment. To address this, upcoming therapies are increasingly combinatorial but bear widely varied results [152, 153, 156, 157] and often are conducted in animal models which bear poor translatability to human studies [186]. The progress made in recent years on understanding GBM stratification and interaction with brain parenchyma as well as immunologically active compartments of the brain is providing a basis for stratified immune therapy defined by the GBM brain tumor subtype; however, therapies have yet to take advantage of this stratification.

An important example of where this could occur is in the treatment of recurrent glioblastoma. At present CAR T cell therapy is often reserved for relapsed/recurrent tumors [154] which are characterized by tumor subtype switching to a mesenchymal state that is notably refractory to radio and temozolomide therapy and bears a highly immunosuppressive TME [48]. EGFRvIII is most widely expressed in the classical tumor subtype [187] which is poorly represented in recurrent GBM [188], therefore, the lack of efficacy of EGFRvIII CAR T cells in these tumors may be explained by a decrease in subtype expression. This can also be reflected in Greenwald et al.*'*s [55] model of GBM stratification where recurrence is likely to occur at the hypoxic core [189] and thus adopt a hypoxia‐related mesenchymal signature before stratifying into proneural and classical subtypes at the tumor edge.

Hypoxia addressing therapies suffer from lack of efficacy which may be explained by the availability to the hypoxic core. Fluid clearance studies in the brain indicate that the mesenchymal core has significantly slower interstitial fluid clearance than surrounding GBM tumor, indicating a potential shielding effect that may prevent penetrance of antibody‐mediated therapies [190]. Further characterization of the GBM environment may thus provide therapeutic mechanisms that could be tailored to specific subtypes of the cancer rather than the tumor as a whole. A further extension of this idea is the stratification of GBM based on tumor location. At present, if the GBM tumor is accessible, then it is targeted with the same therapeutic intervention regardless of location. A tumor developing proximal to the lateral ventricles and thus adjacent to the NSC generating subventricular zone may have a significantly altered tumor environment and organization based entirely on the abundance of NSC‐derived proneural GSC cells.

Furthermore, the rapid understanding of glioblastoma‐brain parenchyma interaction may provide novel avenues for treatment responses. Recently, it has been shown that glioblastomas highly interact with glutaminergic neurons which, in turn, support their development and invasive potential [191]. The neuron immune influence has not been thoroughly investigated in CNS tumors, despite evidence suggesting that neuron interaction may induce immunosuppression in peripheral tumors [192]. Similarly, the formation of tertiary lymphoid structures, which act as lymphocyte refuges in solid tumors such as glioblastoma, is poorly explored outside of presence/absence and formation [193, 194, 195]. Determining the factors inducing formation of these structures and interrogating the prognostic effect of their location within the tumor and the leptomeninges may provide avenues for long‐term therapy responses.

## 7 Conclusion

In summary, despite considerable advancements in understanding the complex biology and microenvironment of glioblastoma, effective and durable therapeutic options remain elusive. Current immunotherapies and hypoxia‐targeting strategies, while promising in other malignancies, have thus far shown limited benefit for GBM patients due to unique tumor heterogeneity, adaptive resistance, and the challenging tumor microenvironment. Future directions must focus on the integration of molecular and spatial tumor stratification, improved delivery methods, and a deeper exploration of the tumor's interaction with its neural and immune context. This approach may unlock more precise, personalized treatments and ultimately improve outcomes for individuals facing this aggressive cancer.

## Author Contributions

**Amit Sharma:** writing – review and editing. **Adrian A. Achuthan:** writing – review and editing, supervision. **Emerson Achari:** writing – original draft. **Jarek Maciaczyk:** writing – review and editing. **Ingo G. H. Schmidt‐Wolf:** writing – review and editing. **Farah Ahmady‐Nield:** writing – review and editing. **Rodney B. Luwor:** writing – review and editing, supervision.

## Conflicts of Interest

The authors declare no conflicts of interest.

## Data Availability

Data sharing not applicable to this article as no datasets were generated or analysed during the current study.
